# Loot box spending is associated with problem gambling but not mental wellbeing

**DOI:** 10.1098/rsos.220111

**Published:** 2022-08-17

**Authors:** Peter J. Etchells, Alexandra L. Morgan, Daniel S. Quintana

**Affiliations:** ^1^ Psychology Centre for Health and Cognition, Bath Spa University, Bath BA2 9BN, UK; ^2^ Department of Psychology, University of Oslo, 0315 Oslo, Norway; ^3^ NevSom, Department of Rare Disorders, Oslo University Hospital, Norway; ^4^ Norwegian Centre for Mental Disorders Research (NORMENT), University of Oslo and Oslo University Hospital, Norway

**Keywords:** video games, loot boxes, gambling, mental health

## Abstract

There are emerging concerns that loot boxes—digital video game items that can be purchased for a chance at randomized rewards—are associated with problematic gambling behaviours and, in turn, are potentially harmful. Current research suggests consistent correlations between loot box spending (LS) and problematic gambling symptomology; however, little research has looked at relationships with mental wellbeing. Here, we used a Bayesian hypothesis testing framework to assess the relative strength of evidence for relationships between LS, excessive gaming, problem gambling, mental wellbeing and psychological distress. Two thousand seven hundred twenty-eight participants who reported playing games containing loot box mechanics in the past month answered a survey assessing the above measures, as well as other forms of digital spending. The results showed extremely strong evidence for a positive correlation between LS and problem gambling; however, there was no evidence to suggest relationships between such spending and mental wellbeing or psychological distress. Exploratory results suggested that individuals who spend money on loot boxes also spend more across a range of digital purchases generally. The findings highlight an urgent need to understand what constitutes harm when considering LS effects and provide further context for discussions regarding how best to regulate such mechanisms.

## 1. Introduction

The way that video games are marketed and monetized has changed considerably in the last decade, coinciding with an increasing shift towards online modes of play. Traditionally, business models would involve a one-off purchase of a fully encapsulated game, with no further spending mechanisms implemented. Recently, there has been an industry shift towards alternative revenue models wherein the base game (either purchased or provided free of charge) is supplemented by frequent content additions, which are purchased for a small amount of money (known as ‘microtransactions’; [1]). For video games developers, this has the advantage of effectively removing the cap on how much a given game will cost a player; new content can be added indefinitely, thereby creating a much more consistent and long-lasting revenue stream.

However, as such microtransaction models have proliferated, there has been growing concern among researchers, clinicians and policymakers about the types of mechanisms that they employ, and whether they are potentially predatory or harmful in nature (e.g. [2]). One of the most well-known types of in-game microtransaction model involves the use of loot box systems, which have recently been argued to be ‘psychologically akin’ to gambling [3], and have been the focus of attention in a multitude of countries with respect to how they may sit in relation to existing gambling legislation.

Loot boxes are digital items that offer players a random chance to obtain desirable additional game content such as character outfits, weapons, cards, boosts or other digital collectibles and can either be freely obtained via in-game progression or purchased with real money. Depending on the specific game, such items may either confer in-game advantages over other players, or be purely cosmetic in nature. Importantly, there is no one universal mechanism through which loot box systems can be implemented, which creates difficulty in understanding the specific effects that engaging in loot box purchase behaviours can have. For example, in the first-person shooter, Overwatch loot boxes can be earned for free through level progression, or else can be purchased in bulk for anywhere between £1.99 and £34.99 GBP (or $1.99 to $39.99 in the USA), whereas in Star Wars: Battlefront 2 loot boxes cannot be purchased for money and are solely acquired for free through meeting certain requirements as the player progresses. The items acquired from loot boxes in Overwatch are purely cosmetic and confer no in-game advantages, whereas the cards obtained from packs in the online card game Hearthstone offer new ways to play and confer competitive advantages to players. The varied nature of such implementations poses a specific problem for recent efforts to update and improve gambling legislation at a national level, as there is currently some disagreement (e.g. [3–5]) within the research literature as to what extent loot boxes share mechanistic similarities with more traditional forms of gambling, and therefore to what extent they should be regulated as such.

Given that loot box systems are a relatively new addition to the video game ecosystem, extant research on the behavioural impact of loot box systems is necessarily embryonic in nature. While a number of research studies have shown a consistent positive relationship between loot box purchase behaviour and levels of problematic gambling (e.g. [6–10]), the specific nature of this relationship is still unclear. For example, it is currently unknown whether loot box purchase behaviour can cause problematic gambling behaviours to develop, vice versa, or whether a third underlying factor precedes both. Similarly, while loot boxes may in some cases share mechanistic similarities to more traditional forms of gambling, it is not clear that they have the same effects on behaviour, or whether they have different risk and protective factors [4]. Moreover, the vast majority of work in this area has focused on the comparison between loot box systems and traditional forms of gambling, in the particular context of whether or not loot box purchase behaviour impacts on, or is moderated by, risk-taking and self-regulatory behaviours. However, there is currently little research looking at the comparison in the context of any impacts on mental health and wellbeing.

To the best of our knowledge, only one study has looked at this relationship within the context of psychological distress. Using a sample of 1049 participants from New Zealand, Australia and the United States, Drummond *et al*. [11] showed that loot box purchase behaviours were weakly associated with both positive and negative shifts in mood, as well as with higher levels of psychological distress. Furthermore, the association between digital game purchase behaviour and problematic gambling symptomology held true not just for loot box purchase behaviours, but for non-randomized game purchase behaviours more generally, a finding in contrast with previous research [8]. This study also suggested an additive effect when excessive gaming behaviour was taken into account. Understanding how loot box spending (LS) behaviours interact with problematic gambling symptomology as well as wellbeing is therefore critical to our understanding of what the profile of potential harm may look like.

Despite the current gap in knowledge regarding the relationship between loot boxes and wellbeing, there are currently discussions in a number of countries regarding how policy changes can be implemented to protect video game users from harm. In some countries (e.g. Belgium), this has already resulted in loot boxes being banned [13], whereas in others (such as the Netherlands), bans have been enacted and then later overturned. In the UK, there are current discussions being held as to how best to regulate such mechanisms [14], and similar discussions are ongoing in Australia and New Zealand, whereas legislation has largely stalled in the USA. Given the urgency of input into policy discussions, there is therefore a need for further research to understand the potential effects that loot box purchase behaviours can have on problematic gambling behaviours and mental wellbeing, and the extent to which these are related to excessive gaming and more traditional forms of gambling.

### 1.1. The current study

Here, we extend our current understanding of loot box effects in a number of ways. The study reports data from 51 countries across the globe and so represents the most wide-spread survey of loot box engagement to date. We examine the relationships between the amount of money that individuals report spending on loot boxes, problematic gambling symptomology and excessive gaming, and further include measures of mental wellbeing and psychological distress. This allows us to assess whether, independent of any relationships with gambling behaviours, LS has a more specific association with wellbeing. The study is also, to the best of our knowledge, the first to assess these relationships within a Bayesian analytical framework. Previous research in the area adopts a frequentist approach, and while this may provide useful information about the existence of relationships between the measures of interest, a conventional frequentist hypothesis testing approach cannot assess the evidence for the null hypothesis, relative to an alternative hypothesis. Importantly, this provides an opportunity to falsify hypotheses and to quantify the extent to which data support the null hypothesis over the alternative, or vice versa (see [15]. Adopting a Bayesian approach therefore has the potential to add significant value to our understanding of the extent to which any associations between LS, gambling and wellbeing are meaningful. As such, we made a number of preregistered predictions before data collection, which can be found in the preregistration here (doi:10.17605/OSF.IO/2ZHRM). We initially posed 27 hypotheses for consideration, but following peer review, it was decided that for the purposes of parsimony, we condense these into a smaller number of key hypotheses of interest which we outline below. The rationale for this, which was discussed and agreed upon by all authors, was to simplify the exhaustive approach specified in the preregistration and amalgamate original hypotheses into clusters aligned with spending categories. As such, the hypotheses specified below do not practically differ from those originally specified, but represent a reorganization for ease of interpretation. Additionally, we opted to not report the outcomes for hypotheses based on (i) traditional gambling spend and (ii) categorization of problem gambling scores here as they are not strictly relevant to the core research question. The analyses for the original 27 hypotheses as specified in the preregistration can be found in electronic supplementary material, file S1. In the interests of transparency, 48% (13 out of 27) of these hypotheses were supported, and 30% (3 out of 10) of the revised hypotheses were supported. Here then, we focus on three core areas of interest and report nine of these preregistered hypotheses:

#### 1.1.1. Hypotheses related to spending behaviours

We predicted that LS would

(1) Correlate positively with problematic gambling and disordered gaming symptomologies.
(2) Correlate negatively with mental wellbeing.
(3) Correlate positively with psychological distress.

We predicted that non-gambling, game-related spending would

(4) Not correlate with problematic gambling or disordered gaming symptomologies.
(5) Correlate positively with mental wellbeing.
(6) Not correlate with psychological distress.

We predicted that expenditure on other types of digital purchase would

(7) Not correlate with problematic gambling or disordered gaming symptomologies.
(8) Correlate positively with mental wellbeing.
(9) Not correlate with psychological distress.

A further, non-preregistered hypothesis was tested which related to the relationship between mental wellbeing and LS:

(10) The relationship between mental wellbeing and LS would be moderated by disordered gaming and problematic gambling symptomologies, as well as levels of income and disposable income (DI).

## 2. Methods

### 2.1. Preregistration

The study was preregistered on the Open Science Framework and can be accessed at doi:10.17605/OSF.IO/2ZHRM. The preregistration plan includes all details regarding data collection methods, data exclusion criteria and analysis plans.

### 2.2. Design

The study used a cross-sectional, between-subjects correlational design using an online survey, written in English, hosted by OnlineSurveys. The primary measures were problematic gambling symptoms, measured via the problematic gambling severity index (PGSI; [16]); internet gaming disorder (IGD) symptomology measured by an adapted version of the Internet Gaming Disorder Checklist used by Przybylski *et al*. [17] and Drummond *et al*. [11]; self-reported monthly income (MI) and DI; self-reported LS, traditional gambling activity spending, in-game non-loot box spending, non-gambling digital spending over the past month; mental wellbeing as measured by the Warwick Edinburgh Mental Wellbeing Scale (WEMWBS; [18]); and psychological distress, measured by the Kessler Psychological Distress Scale (K10; [19]). Additionally, secondary, non-preregistered measures were administered for future research purposes and therefore not used in the further analysis here: participants were asked to name the game they most frequently opened loot boxes in, which platform they play this game on, and on average, how much time they spent playing the game each day. They were also asked a series of questions about their loot box habits based on variables used previously by Zendle *et al*. [10], as well as questions about traditional forms of gambling they may engage in. Finally, the Risky Loot Box Index [6] was also included.

### 2.3. Participants

An *a priori* power analysis was conducted using Bayes Factor design analysis [20], and based on the correlational analyses we intended to conduct. Assuming an expected correlation of 0.1, the sample size determination for a fixed-N design was 2470 at 90% power. This analysis implied rates of 5% for inconclusive results and 0% for false negatives.

Participants were a self-selecting sample of video game players aged 18 or over, recruited via social media advertisement (Reddit and Twitter), Prolific and through advertisement via articles published in Science Focus magazine. A total of 11 508 participants initially responded to the call, with 3608 participants completing the survey. Of these, 880 participants were excluded based on the decision criteria outlined in the preregistration document, leaving a final total of 2728 responses for analysis. The preregistered plan was to collect data from 5000 participants; the first 2500 would be selected into the primary test group, with the following 2500 reserved as a replication group. As we were unable to collect enough data to do this, we therefore deviated from the original plan by using all 2728 responses in the primary test group; no replication was conducted.

Participants were recruited from a total of 51 countries; of these, the five most represented were the UK (660 participants, 24.2% of the sample), Poland (428, 15.6%), Portugal (396, 14.5%), the USA (279, 10.2%) and Italy (180, 6.7%).

A total of 1990 participants (72.9%) described themselves as male and 706 (25.9%) as female. A further 32 (1.2%) described their gender as ‘other’; of these, 21 individuals (0.8%) described themselves as non-binary. The mean reported age was 27.9 years (s.d. = 9.0, range = 18–77). Nearly half of the sample (1251; 45.9%) were aged 18–24; 592 (21.7%) were aged 25–29; 363 (13.3%) were aged 30–34; 224 (8.2%) were aged 35–39; 298 (11.0%) were aged 40 and above. This spread is broadly similar to other reported samples in the literature [21,22]. During data collection, all questions on the online survey were mandatory; as such it was impossible to skip answers. Where participants did not complete the survey, no data were collected at all. As such, no missing data are reported.

### 2.4. Measures

#### 2.4.1. Problematic gambling symptomology

Problematic gambling symptoms were measured using the PGSI. This has been shown to be useful for measuring problematic gambling symptomology in non-clinical populations [23]. The index consists of a nine-item questionnaire with scores ranging from 0 to 27. As well as being used as a continuous measure, the PGSI can also be used to categorize participants into four discrete categories: non-problem gamblers (those who score 0 on the index), low-risk gamblers (those with a score of 1–2), moderate-risk gamblers (those who score 3–7) and problem gamblers (those with a score of 8 or more). Internal reliability of this scale was high (Cronbach's *α* = 0.86).

#### 2.4.2. Gaming disorder symptomology

Problematic gaming use was assessed using an adapted version of the IGD Checklist [17], as used in recent work by Drummond *et al*. [11]. The checklist consists of a nine-item questionnaire based on the diagnostic criteria for IGD proposed in DSM-V, with scores ranging from 9 to 36 and higher scores indicating more severe symptoms of excessive gaming. Internal reliability of this scale was high (Cronbach's *α* = 0.84).

#### 2.4.3. Mental wellbeing

Mental wellbeing was assessed using the WEMWBS [18]. The questionnaire consists of 14 items which relate to an individual's state of mental wellbeing over the preceding two weeks, with scores ranging from 14 to 70. Internal reliability of this scale was high (Cronbach's *α* = 0.92).

#### 2.4.4. Psychological distress

Psychological distress was assessed using the Kessler-10 Psychological Distress Scale [19]. This questionnaire consists of a 10-item scale, with scores ranging from 10 to 50 (higher scores indicate greater levels of psychological distress). Internal reliability of this scale was high (Cronbach's *α* = 0.92).

#### 2.4.5. Monetary measures

A number of self-report questions were used to gather data on income and spending habits over the past month. We asked participants to report, in their country's currency, approximately how much they earned and how much DI they had. DI was defined as the money participants have remaining after taxes and necessary expenditures such as mortgage/rent, rates, loan repayments and food. In terms of spending habits, four questions were asked regarding how much was spent in the last month: participants were asked to report approximately how much they spent on loot boxes, on traditional gambling activities, on non-gambling-related digital video game purchases and finally on non-gambling, non-video game purchases (for example, digital music, apps, ebooks and computer software). All monetary measures were converted into British pounds on 21 May 2021, using Google currency data, provided by Morningstar for Currency on that date.

### 2.5. Data analysis

In the following analyses, we adopted a descriptive classification scheme for interpreting Bayes factors based on that suggested by Lee & Wagenmakers [24]. Using this scheme, a BF_10_ > 100 constitutes extreme evidence for the alternative hypothesis. A BF_10_ > 30 indicates very strong evidence, BF_10_ > 10 strong evidence, BF_10_ > 3 moderate evidence and BF_10_ > 1 anecdotal evidence. Conversely, a BF_10_ < 0.01 indicates extreme evidence in favour of the null hypothesis, BF_10_ < 0.03 very strong evidence favouring the null, BF_10_ < 0.1 strong evidence, BF_10_ < 0.33 moderate evidence and BF_10_ < 1 anecdotal evidence. Analyses were conducted using JASP, and the analysis file used is available on OSF (doi:10.17605/OSF.IO/2ZHRM).

## 3. Results

### 3.1. Descriptive statistics

**Figure 1.**
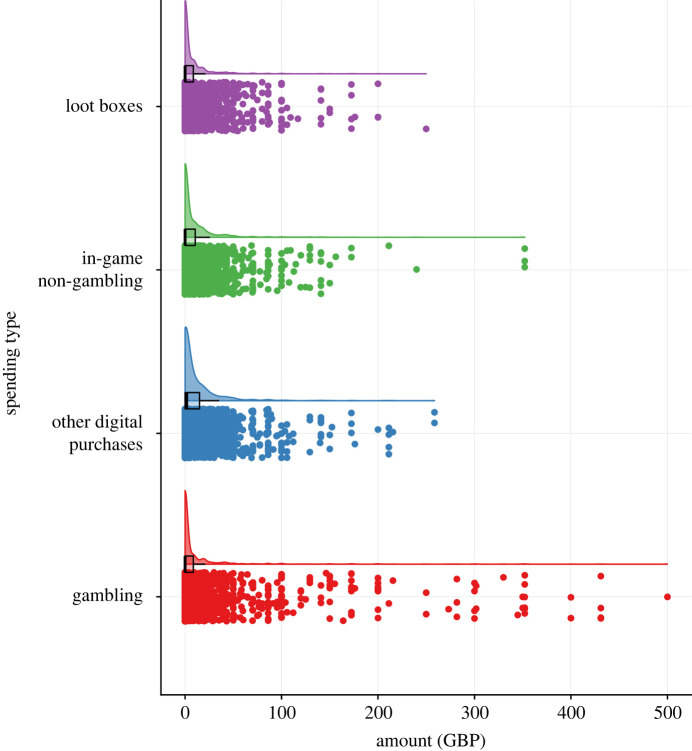
A raincloud plot showing the data distribution and jittered raw data of reported amounts spent for the four measures of spending behaviour.

In line with previous research [11], the amount of money that participants reported spending on loot boxes showed high levels of skewness (5.10) and Kurtosis (36.17). This was true of all measures of spending behaviour and income (Skewness range: 4.37–6.47; Kurtosis range: 27.50–83.02). In general, this appeared to be a result of a large number of respondents reporting low or nil spends and is highlighted in figure 1 below.

Overall, mean amounts reported on LS, in-game non-gambling spending and other digital purchase spending were generally quite low, ranging from £8.06 to £11.81. When spending levels were looked at as a function of PGSI classification, the ranges of spends reported were still relatively low in these three categories, with traditional gambling spend showing the highest and widest spread of reported amounts (table 1).

**Table 1.** Means (s.d.) for the four key measures of spending (in GBP), as a function of PGSI classification (non-problem, low-risk, moderate-risk and problem gamblers).

	loot box spend	in-game non-gambling spend	other digital purchase	gambling spend	*N*
total sample	8.06 (19.63)	9.68 (22.91)	11.81 (23.59)	13.40 (43.29)	2728
non-problem gamblers	5.15 (13.87)	9.12 (23.42)	10.87 (23.07)	4.20 (16.89)	1522
low-risk gamblers	9.41 (19.36)	9.73 (22.31)	11.04 (20.88)	12.08 (32.20)	645
moderate-risk gamblers	12.27 (26.28)	11.02 (22.82)	15.12 (27.27)	32.23 (71.70)	451
problem gamblers	23.21 (38.13)	11.65 (19.41)	15.67 (27.83)	71.15 (97.57)	110

**Figure 2.**
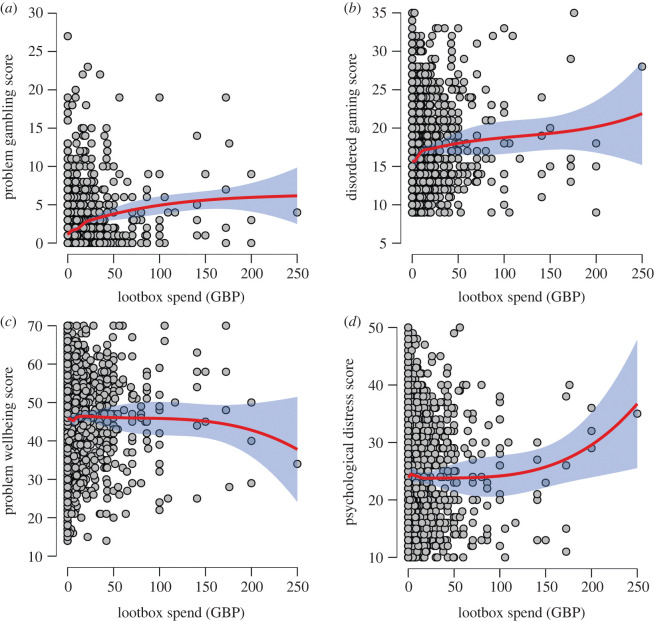
Scatterplots of LS and problem gambling (*a*), disordered gaming (*b*), mental wellbeing (*c*) and psychological distress (*d*) scores. Red lines denote smoothed regression lines and shaded blue areas denote 95% confidence intervals.

Figure 2 shows the relationships between LS and the four key behavioural and wellbeing measures. Given the relatively large number of participants reporting small amounts of LS, the general trends for each association were broadly flat save for large spend amounts. At these levels, the trends appeared to follow the expected directions in line with our hypotheses (for example, an increasingly negative association between LS and mental wellbeing, and an increasingly positive association with psychological distress); however, the concurrent increase in the margin of error means that these trends should be interpreted with extreme caution.

### 3.2. Correlational analyses

Bayesian correlations were used to assess the relationships predicted in Hypotheses 1–9. As a result of the high levels of skewness and Kurtosis in the spend data, as per the registered analysis plan, correlations were assessed using Kendall's Tau-B in place of Pearson's *r*. The priors for each analysis were described by a beta distribution centred around zero and with width parameters based on reasonable estimations based on prior literature. For the relationships between LS and problematic gambling and disordered gaming symptomologies, we used estimates of the expected correlation of 0.26 and 0.25 respectively, based on a meta-analysis by Garea *et al*. [25]. For the relationship between LS and psychological distress, we estimated that the expected correlation would be 0.17, based on previous work by Drummond *et al*. [11]. Drummond *et al*.'s study also included a measure of positive mood, and we used this to estimate an expected correlation between LS and mental wellbeing of 0.15. As a result, the four prior width parameters we used were 0.05 (for LS/problem gambling), 0.048 (for LS/disordered gaming), 0.0164 (for LS/mental wellbeing) and 0.0213 (for LS/psychological distress). These parameters assume a 90% confidence level that the observed correlation coefficient lies within the ranges specified. In the absence of data from previous literature regarding the other spending types, we opted to use the same prior widths as above for the analyses relating to non-gambling in-game spending and other digital purchase spend. The use of a variety of prior widths was a deviation from our preregistered protocol. However, we also applied robustness checks for a range of stretched prior widths from 0.0001 to 2, which evaluated the sensitivity of the prior width on Bayes Factors. For all cases, the robustness checks indicated that the Bayes Factors reported below remained relatively stable for a wide range of stretched prior widths, save for extremely small values.

Table 2 shows the relationships between the three measures of spending behaviour, problem gambling and disordered gaming symptomologies, mental wellbeing and psychological distress, as assessed by Hypotheses 1–9.

**Table 2.** Kendall's Tau correlations between problem gambling scores, disordered gaming scores, loot box spend, gambling spend, non-gambling game-related spend and other digital purchase spend.

	problem gambling	disordered gaming	mental wellbeing	psychological distress
loot box spend	0.201^a^	0.105^b^	0.013	0.007
non-gambling game-related spend	0.074^b^	0.173^b^	−0.028	0.046^a^
other digital purchase spend	0.073^b^	0.015	0.029	−0.018

^a^BF_10_ > 10.

^b^BF_10_ > 100.

As can be seen above, Hypotheses 1 was supported, predicting a positive correlation between LS, problem gambling (BF_10_ = 1.85 × 10^52^) and disordered gaming symptomologies (BF_10_ = 9.11 × 10^13^). Hypothesis 2 predicted a negative correlation between LS and mental wellbeing; however, this was not supported: there was strong evidence in favour of the null hypothesis here (BF_10_ = 0.11). Similarly, Hypothesis 3 predicted a positive correlation between LS and psychological distress; again this was not supported, with moderate evidence in favour of the null hypothesis (BF_10_ = 0.31).

Hypothesis 4 predicted no correlation between non-gambling, game-related spending and problematic gambling or disordered gaming symptomologies. This was not supported; our analyses suggested extremely strong evidence for a small correlation between this type of spending and problematic gambling (BF_10_ = 1.55 × 10^6^), and extremely strong evidence for a larger correlation with disordered gaming (BF_10_ = 2.34 × 10^38^). Hypothesis 5 predicted a positive correlation with mental wellbeing, and Hypothesis 6 predicted no relationship with psychological distress. The results of our analyses indicated evidence for the opposite in both cases; there was strong evidence in favour of there being no correlation between non-gambling game-related spending and mental wellbeing (BF_10_ = 0.07), and very strong evidence in favour of there being a small positive correlation between non-gambling game-related spending and psychological distress (BF_10_ = 93.26).

Focusing on the final spending category, there was partial support for Hypothesis 7, in that there was moderate evidence to suggest that digital purchase spend was not correlated with disordered gaming (BF_10_ = 0.25), but counter to the hypothesis, there was extremely strong evidence in favour of a small positive correlation with problem gambling (BF_10_ = 1.01 × 10^6^). Hypothesis 8 predicted a positive correlation with mental wellbeing; there was moderate evidence in favour of this (BF10 = 2.44); however, the correlation was extremely small. Finally, Hypothesis 9 predicted no correlation between other digital purchase spending and psychological distress; here, although the correlation was again very small, there was only anecdotal evidence in favour of the null (BF_10_ = 0.50).

### 3.3. Exploratory analyses

#### 3.3.1. Regression analysis

Bayesian multiple regression was used to assess the relationship between mental wellbeing and LS as hypothesized in H10. The regression included mental wellbeing as the outcome variable, with LS, problem gambling scores, disordered gaming scores, MI and DI as predictor variables. A uniform prior model was assumed, and a Jeffreys–Zellner–Siow (JZS) prior on parameters was used (r-scale = 0.354). Table 3 shows the model comparisons. There was extremely strong evidence for the best model which included DI (*M* = 0.002, 95% credible intervals = 0.001–0.002) and disordered gaming (*M* = −0.51, 95% credible intervals = −0.59 to −0.44) as predictors (BF_10_ = 3.76 × 10^51^), compared to the null model. There was moderate evidence for not including LS as a predictor (BF_inclusion_ = 0.22). 8.8% of the variance in mental wellbeing scores was accounted for by the best model, and including LS only appeared to account for an extra 0.1% of the variability in wellbeing scores.

**Table 3.** Model comparisons for Bayesian multiple regression assessing relationship between mental wellbeing, LS, PGSI disordered gaming symptomology (IGD), MI and DI. The 10 best model comparisons are shown.

models	P(M)	P(M|data)	BF_M_	BF_10_	*R*^2^
null model	0.025	1.81 × 10^−52^	7.04 × 10^−51^	1.00	0.00
DI + IGD	0.025	0.678	82.273	3.76 × 10^51^	0.088
LS + DI + IGD	0.025	0.142	6.452	7.86 × 10^50^	0.089
DI + PGSI + IGD	0.025	0.065	2.725	3.62 × 10^50^	0.088
MI + DI + IGD	0.025	0.061	2.528	3.37 × 10^50^	0.088
LS + DI + PGSI + IGD	0.025	0.022	0.864	1.20 × 10^50^	0.089
LS + MI + DI + IGD	0.025	0.014	0.571	7.98 × 10^49^	0.089
MI + DI + PGSI + IGD	0.025	0.007	0.271	3.82 × 10^49^	0.088
DI + PGSI + IGD + PGSI*IGD	0.025	0.005	0.205	1.37 × 10^49^	0.089
LS + MI + DI + PGSI + IGD	0.025	0.002	0.097	2.89 × 10^49^	0.088

#### 3.3.2. Comparison of loot box spending with non-spending

A notable proportion of respondents (*N* = 1641) indicated that although they had played a game containing a loot box mechanic within the past month, they had not spent any money on loot boxes during this time. As such, we undertook an exploratory analysis comparing participants who reported spending no money on loot boxes with those who had spent some amount. Table 4 provides an overview of the mean scores for each of the key behaviour and wellbeing measures.

**Table 4.** Means (s.d.) for problem gambling, disordered gaming, mental wellbeing and psychological distress scores, separated by loot box spend status.

	problem gambling	disordered gaming	mental wellbeing	psychological distress
gamers who do not spend money on loot boxes	1.07 (2.21)	15.65 (4.89)	45.81 (10.16)	23.91 (8.17)
gamers who do spend money on loot boxes	2.20 (3.41)	16.90 (5.22)	46.09 (10.28)	24.20 (8.59)

A series of Bayesian *T*-tests were conducted to explore the relationship between LS and problem gambling symptoms, disordered gaming symptomology, mental wellbeing, psychological distress and other types of spending. The priors for each analysis were described by a Cauchy distribution centred around zero and with width parameters based on the estimates described for the correlational analyses above. The widths used were: 0.041 for problem gambling, 0.04 for disordered gaming, 0.023 for mental wellbeing and 0.027 for psychological distress. We opted to use the JASP default prior width of 0.707 for the comparisons relating to other types of spending. In line with our other analyses, there was extreme evidence to support a difference, when comparing loot box spenders with non-spenders, in problem gambling scores (*M*: 2.20 versus 1.07; BF_10_ = 2.38 × 10^21^) and disordered gaming symptomology (*M*: 16.90 versus 15.65; BF_10_ = 9.47 × 10^6^). For both mental wellbeing and psychological distress, there was anecdotal evidence favouring the null model (BF_10_s = 0.73 and 0.74, respectively). Regarding other types of spend, there was extreme evidence to support a difference, when comparing loot box spenders with non-spenders, in gambling spend (*M*: £19.52 versus £9.34, BF_10_ = 3.16 × 10^6^) and non-gambling-related video game spend (*M*: £12.65 versus £7.71; BF_10_ = 1.77 × 10^5^), and there was moderate evidence to support a difference for other digital purchase spending (*M*: £13.43 versus £10.73; BF_10_ = 3.06). These results indicate that those who tend to spend money on purchasing loot boxes also spend more money across a range of digital purchases more generally.

## 4. Discussion

The current study investigates the relationship between LS, problem gambling symptoms, mental wellbeing, psychological distress and excessive gaming symptoms across a large sample of participants from numerous countries. To the best of our knowledge, this is the first study to use Bayesian methods in this context. The results show that there is extremely strong evidence to suggest that LS is correlated with both problem gambling symptoms and excessive gaming symptoms, with problem gamblers spending more on loot boxes than those exhibiting fewer symptoms. However, there is no evidence to suggest, relative to an alternative hypothesis, that LS is correlated with either mental wellbeing or psychological distress. Regression analyses indicate that individuals who report higher levels of DI and lower disordered gaming scores tend to have higher levels of mental wellbeing. There was no strong evidence to include LS in the model, and in general, all models tested did not account for much of the variance in mental wellbeing, suggesting that other factors may be more important.

This finding was reinforced via exploratory analyses comparing participants who reported playing games which contained loot box mechanics but did not spend any money on them, versus those who did report spending money. While there is strong evidence to suggest higher levels of problem gambling and disordered gaming symptomologies among loot box spenders, there is no evidence to suggest any differences in mental wellbeing or psychological distress between these groups. Moreover, there is evidence to suggest that individuals who spent more money on loot boxes also spent more money on other types of digital purchase, both gaming and non-gaming in nature, more broadly. It is therefore a question for future study as to whether loot box purchases are indicative of a general impulse control issue when it comes to spending behaviours, or whether they should instead be considered a unique category of spending which carry their own risks.

To date and to the best of our knowledge, only one study has investigated the relationship between LS and indicators of mental wellbeing. Drummond *et al*. [11] found positive correlations between LS and psychological distress, negative mood and also positive mood. Conversely, in the present study, there was no evidence for a correlation between LS and either psychological distress or mental wellbeing—that is, no indication that LS has either a positive or negative effect on mental health. There are a number of reasons why this may be the case. The first is that Drummond *et al*.'s study population appeared to be unrepresentative in some respects—for example, in their sample, there was an over-representation of female respondents, and the proportion of self-reported problem gamblers was much higher than expected. In the present study, the number of female respondents was much smaller (although this was smaller than the proportion in the general gaming population), as was the proportion of problem gamblers (although this was still slightly higher than would be expected in the general population). The second reason is that the absolute amount of spending reported by participants was relatively small, and combined with sample differences may have contributed to reported variation in what are likely to be very mild associations with wellbeing. Third, it is possible that the effects may be the result of a false negative.

Our results concerning the relationship between LS and problem gambling concur with previous studies which show a clear relationship between the two. In line with Drummond *et al*. [11], participants classified as problem gamblers spent more on loot boxes than moderate or low-risk gamblers, who in turn spent more than non-problem gamblers (see electronic supplementary material, S1). Across all categories of spending, problem gamblers tended to show quite a high degree of variability in reported spends, suggesting that there is a small subset of individuals within this category who exhibit issues with controlling their spending behaviour, which is exacerbated when the type of purchase activity involves an element of gambling. However, the average loot box spend for problem gamblers was only around £34, which suggests that the financial impact of problematic gambling behaviours when it comes to purchasing loot boxes is relatively small. This finding is in line with previous estimates from the literature, which estimate the typical loot box spend among problem gamblers to £30–£40 per month [11].

The results of the regression analyses further suggest that the relationship between mental wellbeing and LS is weak, as the best-fitting model did not include LS as a predictor: the only predictor variables that were included were DI and disordered gaming. In general, the models were a poor fit to the data, with the above measures accounting for very little of the overall variance in mental wellbeing scores. Taken together with the fact that participants in the present study spent relatively little on loot boxes and did not appear to show problems in mental wellbeing, it is not currently clear that we have a deep understanding of what an ‘at-risk’ user profile for high loot box engagement might actually mean or look like. Where some have suggested that considering such engagement within a gambling framework is most appropriate [26], others argue that it is better to couch it within a framework focusing on excessive gaming [27]. However, both approaches are considerably underdeveloped with respect to loot box effects. Going forward, it is clear that new theoretical frameworks need to be developed which more appropriately encapsulate and represent problematic or excessive gaming, which can then be used to develop a deeper understanding of the relationship between that, problem gambling, and loot box purchasing.

The present results further need to be considered within the context of the timeframe in which the data were gathered. The data were collected between October 2020 and May 2021, during which time various public health measures were in place across the globe to curb the effects of the COVID-19 pandemic. It has been argued that concerns about the impact of the pandemic, coupled with country-specific stay-at-home orders of varying restrictiveness, may have resulted in video game players becoming more socially isolated and therefore engaging in playing behaviours more intensively. In turn, this may have resulted in an increase in engaging in LS behaviours during this time. Work by Hall *et al*. [28] found that while contamination concern was associated with more excessive gaming and risky loot box engagement, there was little evidence to suggest that pandemic-related isolation resulted in greater levels of LS. They also note that the effects of pandemic-related isolation may artificially increase the effect size of gaming-related effects. It therefore may be the case that the strength of the relationships between problem gambling and LS shown in the present study may be larger than would otherwise be expected. We echo Hall *et al*.'s comments that caution is urged in interpreting the findings from studies on media effects conducted during the pandemic, as they may not be representative of behaviour during non-pandemic periods.

One apparent limitation of the study is that while all participants had played a game containing loot box mechanics in the past month, a large number reported that they spent no money on them. However, this is useful data in and of itself; that so many players report that they do not spend money in such games suggests that the presence of loot box mechanisms within a game is not guaranteed to be associated with problem gambling behaviours or wellbeing. We undertook exploratory analyses to assess potential differences between participants who did and did not report spending money on loot boxes. In line with our preregistered prediction, there was extremely strong evidence here to support differences in problem gambling symptomology depending on spend status; individuals who spent money on loot boxes also reported higher levels of problem gambling scores. However, there were again no differences between these two groups in terms of mental wellbeing or psychological distress. Interestingly, individuals who reported spending money on loot boxes also reported spending more money on other types of digital purchase more broadly, compared to those who reported not spending money on loot boxes. This suggests that LS is not necessarily a special category of purchase behaviour and may indicate that individuals who score highly in terms of problem gambling symptomology may have a general difficulty in controlling spending. Given their exploratory nature, we would urge care in interpreting these results; however, it is clear that further research needs to assess how problem gambling symptomology impacts on non-gambling digital purchase behaviours.

There is an emerging body of research that is attempting to unpick the relationship between LS behaviours and potential harm to the user (e.g. [6–12]). The present study adds to the general body of evidence suggesting that there is a clear positive correlation between such spending behaviours and problem gambling symptomologies. However, our results also suggest that we need to take considerable care in terms of the extent to which the current body of evidence can meaningfully inform national policy. As already noted, while it is clear that there is a correlational relationship between problem gambling symptoms and LS, we are still to develop a clear understanding of if or how this translates into a demonstrable effect on wellbeing. The present study calls into question the idea that there is a simple or clear-cut relationship between increasing loot box use and harm, in the context of mental wellbeing, and as such we urge caution in implementing hard-line policies or regulations on the basis of assuming otherwise. For example, there have been a number of calls for harm minimization techniques such as the implementation of spending limits [26], or limiting the number of loot boxes that can be purchased per day [29]. However, it is known from other work that artificially restricting access to desirable items can have the opposite effect than intended, in that individuals are more likely to want to spend more later (e.g. [30]). Urgent research is clearly needed to fully elucidate the causal relationships between LS, behaviour and wellbeing, and this can be best achieved by independent researchers being given appropriate access to relevant industry data. Therefore, while we make no recommendations as to what harm-reduction policy in this area should look like, we reiterate numerous calls by researchers in the field for policymakers to enable and facilitate interactions between researchers and industry, so that the field no longer has to reply on self-report measures.

While the present study informs our understanding of the relationship between LS and wellbeing, there are a number of limitations which are worth considering. The study reports a cross-sectional study and as such, we cannot infer causal relationships. As such, it is still not clear whether LS leads to problem gambling symptoms, or vice versa. This remains a priority question for the field and, as noted above, needs the cooperation of industry in order to fully answer. Further, it is worth noting that the study is based on self-report measures, and within the context of technology use, there have been recent studies to suggest that such measures are not completely reliable in determining accurate estimates of usage [31]. While these studies largely focus on estimates of the amount of time spent on screen-based activities (of which video game play is one), it is reasonable to apply caution on comparable self-report measures of monetary expenditure within games. Further research is needed to assess the reliability of such measures. An additional consideration is the large number of tests performed, which in a frequentist context can raise the risk of false positives if there is no correction for multiple tests. However, this is not a concern when using a Bayesian approach as long as an appropriate prior distribution is used [32].

In conclusion, the present study supports the current body of evidence which suggests that there is a positive correlation between problem gambling symptomology and the amount of money spent on loot boxes. Individuals who also score more highly on measures of disordered gaming symptomology are also more likely to spend more. However, this does not appear to translate into a clear harm in terms of wellbeing, as we found no associations between LS and either mental wellbeing or psychological distress. Therefore, while our findings are in line with previous work suggesting that there is a small proportion of video game players for whom engaging in LS may be detrimental, it is currently not clear whether this translates into some form of demonstrable harm (financial or otherwise), if at all. Before concrete policy recommendations can be enacted, further studies should focus on developing an appropriate theoretical framework within which to consider loot box effects, and more specifically consider what the user profile for at-risk individuals looks like. This requires careful definition and operationalization of harm, as well as new and robust mechanisms for the gaming industry to responsibly share data with independent scientists.

## Data Availability

The data and associated JASP analysis code are available on OSF: https://osf.io/2zhrm/. Electronic supplementary material is available online at [33].
